# Association Between Physician Online Rating and Quality of Care

**DOI:** 10.2196/jmir.6612

**Published:** 2016-12-13

**Authors:** Kanu Okike, Taylor K Peter-Bibb, Kristal C Xie, Okike N Okike

**Affiliations:** ^1^ Department of Orthopedic Surgery Kaiser Permanente Moanalua Medical Center Honolulu, HI United States; ^2^ University of Colorado Boulder, CO United States; ^3^ Iolani School Honolulu, HI United States; ^4^ Department of Patient Experience University of Massachusetts Memorial Healthcare Worcester, MA United States

**Keywords:** online reviews, cardiac surgery, physician quality

## Abstract

**Background:**

Patients are increasingly using physician review websites to find “a good doctor.” However, to our knowledge, no prior study has examined the relationship between online rating and an accepted measure of quality.

**Objective:**

The purpose of this study was to assess the association between online physician rating and an accepted measure of quality: 30-day risk-adjusted mortality rate following coronary artery bypass graft (CABG) surgery.

**Methods:**

In the US states of California, Massachusetts, New Jersey, New York, and Pennsylvania—which together account for over one-quarter of the US population—risk-adjusted mortality rates are publicly reported for all cardiac surgeons. From these reports, we recorded the 30-day mortality rate following isolated CABG surgery for each surgeon practicing in these 5 states. For each surgeon listed in the state reports, we then conducted Internet-based searches to determine his or her online rating(s). We then assessed the relationship between physician online rating and risk-adjusted mortality rate.

**Results:**

Of the 614 surgeons listed in the state reports, we found 96.1% (590/614) to be rated online. The average online rating was 4.4 out of 5, and 78.7% (483/614) of the online ratings were 4 or higher. The median number of reviews used to formulate each rating was 4 (range 1-89), and 32.70% (503/1538) of the ratings were based on 2 or fewer reviews. Overall, there was no correlation between surgeon online rating and risk-adjusted mortality rate (*P*=.13). Risk-adjusted mortality rates were similar for surgeons across categories of average online rating (*P*>.05), and surgeon average online rating was similar across quartiles of surgeon risk-adjusted mortality rate (*P*>.05).

**Conclusions:**

In this study of cardiac surgeons practicing in the 5 US states that publicly report outcomes, we found no correlation between online rating and risk-adjusted mortality rates. Patients using online rating websites to guide their choice of physician should recognize that these ratings may not reflect actual quality of care as defined by accepted metrics.

## Introduction

Consumers have long used reviews of goods and services to inform their choices. Recently, these trends have spread to the health care arena in the form of online physician review websites [1-14]. According to a recent survey, 65% of respondents were aware of physician rating websites, and 35% had sought online physician reviews within the past year [15]. The survey also found that these online reviews were influential: among those who sought physician ratings information online, 35% reported selecting a physician based on good ratings and 37% reported avoiding a physician with bad ratings [15].

While patients are increasingly using physician review websites to find “a good doctor,” it remains unclear whether online physician ratings actually reflect quality of care. Segal et al analyzed online ratings in relation to surgeon case volume, which they considered to be a proxy for quality of care, and found no correlation between numerical rating and number of procedures performed [13]. Similarly, Gao and colleagues analyzed ratings from the RateMDs.com website in comparison with data obtained from the Virginia Board of Medicine, and found no correlation between physician rating and malpractice claims [8]. However, to our knowledge, no prior study has examined the relationship between online ratings and an accepted measure of quality.

The purpose of this study was to assess the degree to which online physician ratings reflect quality of care. In the US states of New York, New Jersey, Massachusetts, Pennsylvania, and California—which together account for over one-quarter of the US population [16]—risk-adjusted mortality rates are publicly reported for all cardiac surgeons. By analyzing the online ratings of these surgeons in comparison with their clinical outcomes, we sought to assess the degree to which online ratings correlate with quality of care.

## Methods

In June 2015, we accessed the cardiac surgeon “report cards” for all 5 states that publicly report risk-adjusted cardiac surgery mortality rates (ie, California [17], Massachusetts [18], New Jersey [19], New York [20], and Pennsylvania [21]). From the online reports, we recorded the names of all cardiac surgeons practicing in these states, as well as their institutions. For each surgeon listed, we also recorded the 30-day risk-adjusted mortality rate following isolated coronary artery bypass graft (CABG) surgery.

To calculate the risk-adjusted mortality rate, the observed mortality rate is divided by the expected mortality rate and then multiplied by the statewide mortality rate. (For reference, the observed mortality rate is the observed number of deaths divided by the total number of cases, and the expected mortality rate is the sum of predicted probabilities of death for all patients divided by the total number of patients.)

For each surgeon listed in the state reports, we conducted Internet-based searches between July and September 2015 to determine his or her online rating(s). Searches were conducted using surgeon name, location, and specialty. For each online rating identified, we recorded the name of the website, the overall rating, and the number of reviews used to formulate the rating. Online ratings were out of 5. The individuals performing these searches (TKPB and KCX) were blinded to the surgeons’ clinical outcomes.

We assessed the association between surgeon online rating and risk-adjusted mortality rate using the Pearson correlation coefficient. In addition, surgeons were grouped on the basis of average online rating, and risk-adjusted mortality rates were compared using the Student *t* test. Surgeons were also grouped on the basis of risk-adjusted mortality rate quartile, and online ratings were compared using Student *t* test. *P*<.05 was considered statistically significant, and all tests were 2-sided. Statistical analysis was performed using SAS version 9 (SAS Institute Inc).

## Results

There were 614 cardiac surgeons with risk-adjusted mortality rates listed in the 5 state reports (California: 271; Massachusetts: 52; New Jersey: 36; New York: 135; and Pennsylvania: 120). For all states combined, the average 30-day risk-adjusted mortality rate after isolated CABG surgery was 1.68% (SD 1.98, median 1.22%, range 0.00%-16.98%).

We found 96.1% (590/614) of the surgeons to be rated online, including from Healthgrades (n=540) [22], Vitals (n=495) [23], UCompareHealthCare (n=366) [24], and RateMDs (n=103) [25]. We found that 74 of the surgeons were rated on a single website, while 170 were rated on 2 websites, 266 were rated on 3 websites, and 80 were rated on 4 or more websites. The average online rating for the cardiac surgeons was 4.4 on a scale of 1-5, with 1 being the lowest score and 5 being the highest score obtainable. As Table 1 shows, 78.7% (483/614) of the scores were 4 out of 5 or better. The median number of reviews per surgeon was 4, with a wide range (1-89 reviews).

**Figure 1:**
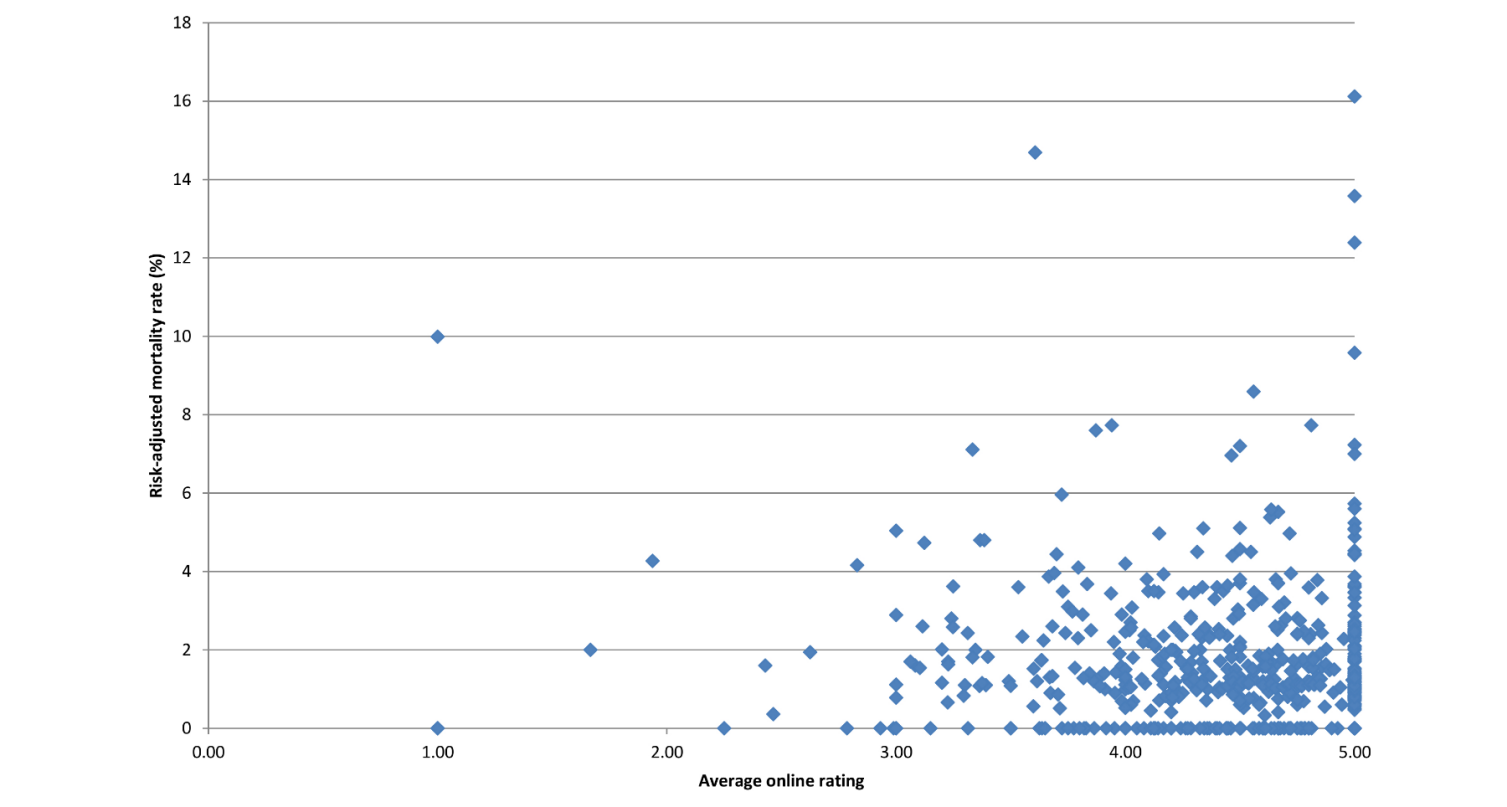
Average online rating versus risk-adjusted mortality rate. Ratings are out of 5.

**Figure 2:**
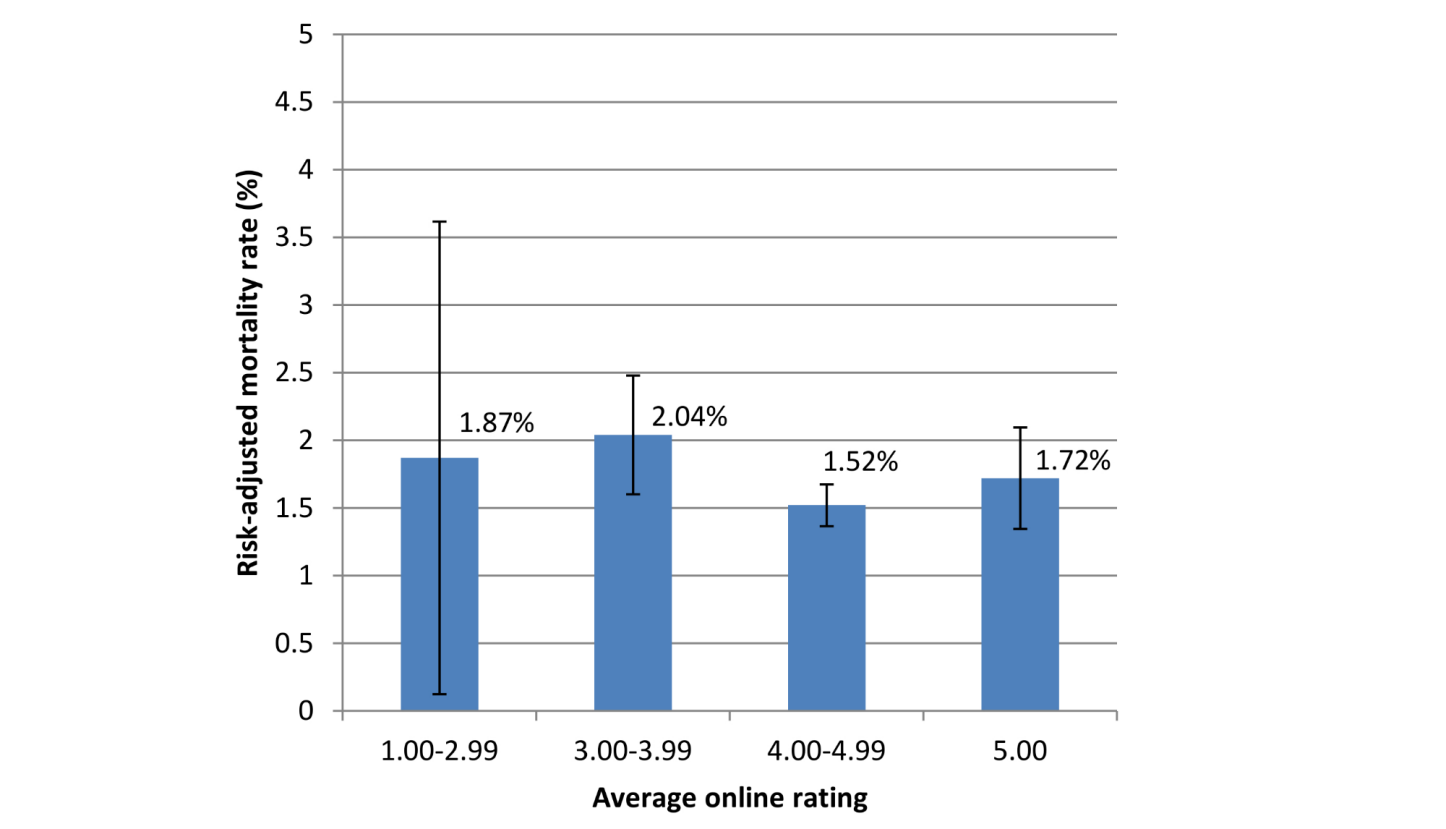
Risk-adjusted mortality rate, by average online rating. Note that the categories of average online rating differ in size. Error bars indicate 95% CIs, which vary in magnitude due to the number of ratings in each category (n=13 for 1.00-2.99, n=94 for 3.00-3.99, n=324 for 4.00-4.99, and n=159 for 5.00). Ratings are out of 5. There were no significant differences between the groups (*P*>.05).

Figure 1 depicts a scatterplot of surgeon risk-adjusted mortality rate versus average online rating. Surgeon online rating did not correlate with risk-adjusted mortality rate (Pearson correlation coefficient –.06, *P*=.13). Risk-adjusted mortality rates were similar for surgeons across categories of average online rating (*P*>.05; Figure 2). Similarly, surgeon average online rating was similar across quartiles of surgeon risk-adjusted mortality rate (*P*>.05; Table 2).

**Table 1:** Average online ratings^a^ of cardiac surgeons who had risk-adjusted mortality rates listed, July-September 2015.

Average online rating	n (%)
5.00	159 (25.9)
4.00-4.99	324 (52.8)
3.00-3.99	94 (15.3)
2.00-2.99	8 (1.3)
1.00-1.99	5 (0.8)
Not rated online	24 (3.9)
Total	614 (100.0)

^a^Ratings are out of 5.

**Table 2:** Average online rating, by quartile of surgeon risk-adjusted mortality rate.

Surgeon risk-adjusted mortality rate		n	Surgeon average online rating^a^		*P* value^b^
Quartile	Range		Mean	SD	
Very low	0.00%-0.41%	148	4.4	0.7	(reference)
Low	0.45%-1.20%	147	4.5	0.5	.34
Medium	1.23%-2.31%	148	4.4	0.6	.49
High	≥2.34%	147	4.4	0.7	.40

^a^Ratings are out of 5.

^b^Compared with surgeons with risk-adjusted mortality rates categorized as “very low.”

## Discussion

In this study of cardiac surgeons practicing in the 5 US states that publicly report outcomes, we found no correlation between online rating and risk-adjusted mortality rates.

We are not aware of any prior study assessing the correlation between physician online rating and accepted measures of quality. However, 2 prior studies have examined the relationship between patients’ subjective assessments of care and objective measures of quality. In these 2 studies, both of which were conducted among individuals over the age of 65 years, the subjective ratings given by patients were not found to correlate with the accepted quality measures [26,27].

Our study is not without its limitations. We used 30-day risk-adjusted mortality rates to measure quality, and it is possible that our results could have differed had we examined long-term mortality rates or rates of major morbidity (such as renal failure or stroke). However, 30-day risk-adjusted mortality is the most commonly accepted measure of quality in the field [28]. In addition, since we investigated cardiac surgeons in 5 US states, it is unclear whether the findings can be generalized to other fields of medicine or other locations.

For physicians, who have long argued that online ratings do not reflect clinical competency [29], the results of our study may not be surprising. However, our findings serve as a reminder that the provision of high-quality medical care may not necessarily translate into higher online ratings.

Our study also has important implications for patients. Consumers are increasingly using online reviews to guide their selection of goods and services, and health care is no exception [15]. Based on the results of our study, patients using online rating websites to guide their choice of physician should recognize that these ratings may not reflect actual quality of care as defined by accepted metrics. In contrast, they may be more reflective of factors such as clinic wait times [30] or bedside manner [31].

In summary, this study of cardiac surgeons practicing in the 5 US states that publicly report outcomes found no correlation between online rating and risk-adjusted mortality rates. Patients using online rating websites to guide their choice of physician should recognize that these ratings may not reflect actual quality of care as defined by accepted metrics.

## Abbreviations

CABG: coronary artery bypass graft
